# Influenza in patients with respiratory failure admitted to intensive care units in Poland and the use of extracorporeal respiratory support: a survey-based multicenter study

**DOI:** 10.1186/s12879-021-06672-w

**Published:** 2021-09-15

**Authors:** Jakub Smiechowicz, Barbara Barteczko-Grajek, Barbara Adamik, Jozef Bojko, Waldemar Gozdzik, Malgorzata Lipinska-Gediga

**Affiliations:** 1 Department of Anaesthesiology and Intensive Therapy, Wroclaw Medical University, Borowska 213, 50-556 Wrocław, Poland; 2 Department of Anaesthesiology and Intensive Therapy, Provincial Hospital in Opole, Kosnego 53, 46-020 Opole, Poland; 3 Department of Anaesthesiology and Intensive Therapy, 4th Military Hospital of Wroclaw, Weigla 5, 50-981 Wrocław, Poland

**Keywords:** Influenza, Intensive care, Respiratory failure, Mechanical ventilation, Coinfection, Extracorporeal life support

## Abstract

**Background:**

In Poland, little is known about the most serious cases of influenza that need admittance to the intensive care unit (ICU), as well as the use of extracorporeal respiratory support.

**Methods:**

This was an electronic survey comprising ICUs in two administrative regions of Poland. The aim of the study was to determine the number of influenza patients with respiratory failure admitted to the ICU in the autumn–winter season of 2018/2019. Furthermore, respiratory support, outcome and other pathogens detected in the airways were investigated.

**Results:**

Influenza infection was confirmed in 76 patients. The A(H1N1)pdm09 strain was the most common. 34 patients died (44.7%). The median age was 62 years, the median sequential organ failure assessment (SOFA) score was 11 and was higher in patients who died (12 vs. 10, p = 0.017). Mechanical ventilation was used in 75 patients and high flow nasal oxygen therapy in 1 patient. Extracorporeal membrane oxygenation (ECMO) was used in 7 patients (6 survived), and extracorporeal carbon dioxide removal (ECCO_2_R) in 2 (1 survived). The prone position was used in 16 patients. In addition, other pathogens were detected in the airways on admittance to the ICU.

**Conclusion:**

A substantial number of influenza infections occurred in the autumn–winter season of 2018/2019 that required costly treatment in the intensive care units. Upon admission to the ICU, influenza patients had a high degree of organ failure as assessed by the SOFA score, and the mortality rate was 44.7%. Advanced extracorporeal respiratory techniques offer real survival opportunities to patients with severe influenza-related ARDS. The presence of coinfection should be considered in patients with influenza and respiratory failure.

**Supplementary Information:**

The online version contains supplementary material available at 10.1186/s12879-021-06672-w.

## Background

Each year influenza activity in Europe is high in the autumn and winter months. A significant part of the population is affected by symptomatic viral infections with impact on public health and the economy. Although most influenza infections are mild, about 2% of cases require hospitalization and up to 10% of hospitalized influenza patients may need admission to the intensive care unit (ICU), mostly because of respiratory distress [1, 2]. In those with the most severe respiratory failure the use of extracorporeal techniques of respiratory support is warranted [3]. In Poland the annual number of influenza cases has been increasing in recent years and reached 5.2 million cases in 2018 [4]. Despite a 15% lower incidence of influenza in the 2018/2019 season, the reported mortality was much higher than in previous years [4]. The most serious cases need admittance to the ICU, yet little is known about the number of such cases and the use of extracorporeal respiratory support in this patient population.

In the present study, we investigated the incidence of influenza in patients with respiratory failure admitted to adult intensive care units in two administrative regions of Poland, the Lower Silesia and Opole voivodeships, in the autumn–winter season of 2018/2019. Additionally, we investigated the outcome, the method of respiratory support used, and the presence of other pathogens in the airways on admittance to the ICU.

## Methods

### Study design and population

A retrospective, multicenter, observational survey was conducted in accredited intensive care units in two administrative regions of Poland. The study was approved by the Wroclaw Medical University Bioethics Committee (Decision No. KB—552/2019) and was conducted in accordance with the Declaration of Helsinki. Patient identification was established on an anonymized basis, and informed consent was not considered necessary due to the non-interventional nature of the study. The survey was distributed via e-mail to ICUs in the Lower Silesia (n = 26) and Opole voivodeships (n = 7), for all ICUs registered by the National Health Fund in these administrative regions. All ICUs (n = 33) were located in different hospitals, and all of them were mixed-type ICUs. Twenty units were teaching ICUs and 13 were non-teaching ICUs. Two of the teaching ICUs were located in university hospitals. The total bed capacity of the units was 255 beds. Participation in the survey was voluntary. Supplementary characteristics of participating ICUs are included in Additional file 1: Appendix S1.

The survey concerned adult patients (≥ 18 years) with confirmed influenza and respiratory failure admitted to the ICUs between 1 November 2018 and 31 March 2019, when most (97%) of laboratory confirmed influenza cases in the general population occurred, according to the National Institute of Public Health–National Institute of Hygiene (NIH) reports [4]. Survey was completed after the patients were discharged from the ICU. Investigators included in the study all patients with a confirmed influenza infection. Standard methods based on real-time polymerase chain reaction (RT-PCR) testing, rapid antigen tests and immunoassays described in the recommendations of the Centers for Disease Control and Prevention (CDC) were used depending on availability in the participating hospitals [5].

### Data collection

The survey comprised questions related to demographic information (age, gender), the type of the influenza virus detected, the sequential organ failure assessment (SOFA) score on admittance to the ICU, the method of respiratory support used, and the outcome (survived, not survived). Questions were also asked concerning other pathogens detected in the airways and testing for *Aspergillus* spp. (galactomannan assay, and culture). Data on positive influenza test results were also collected for all patients admitted to the surveyed hospitals during the studied period. Investigators were contacted for missing data, if needed.

The full version of the questionnaire is reported in Additional file 1: Appendix S2.

### Statistical analysis

The data were analyzed with Statistica 12 (StatSoft. Inc. Tulsa, USA). Continuous variables are presented as medians (with the interquartile range between the 25th and 75th percentiles) and categorical variables are presented as numbers and percentages. The distribution was not normal based on the Shapiro–Wilk test; therefore, statistical analysis was performed using a non-parametric test. The Mann–Whitney U test was used for comparison of the continuous variables between survivors and non-survivors. Categorical variables were analyzed using a Chi-square test. The estimated cumulative incidence rate of influenza-associated respiratory failure in ICU-hospitalizations in the studied area was expressed as cases per 100,000 inhabitants annually. The population of the studied regions was 3.9 million inhabitants at the end of 2018, according to Statistics Poland (https://stat.gov.pl). We assumed that the population was closed during the study and that the incidence of influenza-associated respiratory failure was negligible beyond the influenza season. 95% confidence intervals for the incidence rate were calculated. Statistical significance was considered for p < 0.05.

## Results

### Influenza in ICU

Surveys were received from all 33 currently operating ICUs (response rate of 100%). During the study period, patients with respiratory failure and positive test results for influenza were treated in 21 out of 33 participating ICUs (63.6%), with a capacity of 182 beds. An influenza infection was confirmed in 76 patients and the influenza A/H1N1pdm09 strain was found in 40 of them. One patient tested positive for influenza type B virus. 34 patients with influenza died (44.7%).

51% of patients were male and the median age was 62 years (from 19 to 86 years). The median SOFA score on admittance to the ICU was 11 and was significantly higher in patients who died (12 vs. 10 p = 0.017) (Table 1).

**Table 1:** Baseline patient characteristics

	Entire cohort N = 76	Survivors N = 42 (55.3%)	Non-survivors 34 (44.7%)	p value
Age, years	62 (52–74)	62 (50–72)	66 (57–78)	0.251
Gender, male (%)	51	48	53	0.691
SOFA score	11 (9–12)	10 (8–12)	12 (9–14)	0.017

Data are presented as median [IQR] or N (%)

*SOFA* sequential organ failure assessment

All patients required invasive mechanical ventilation except one, where intubation was avoided by means of high flow nasal oxygen therapy (HFNO). Seven patients in three ICUs had venovenous extracorporeal membrane oxygenation (VV ECMO) therapy and two patients in two ICUs had extracorporeal carbon dioxide removal (ECCO_2_R) therapy. Prone position ventilation was used in 16 patients in seven ICUs (Table 2).

**Table 2:** Types of influenza virus and respiratory support in patients admitted to the ICU during 2018/2019 influenza season

Parameter	Patients with influenza		
	Entire cohort	Survivors	Non-survivors
Influenza virus n (%)	76 (100)	42 (55.3)	34 (44.7)
Type A	75 (98.6)	41	34
Subtype A/H1N1pdm09, n (% of A)	40 (53.3)^a^		
Type B	1 (1.3)	1	0
Respiratory support, n (%)			
Use of IMV	75 (98.7)	41	34
Use of HFNO	1 (1.3)	1	0
Prone-position ventilation	16 (21)		
Use of ECMO	7 (9.2)	6	1
Use of ECCO_2_R	2 (2.6)	1	1

Detailed information for subtype A/H1N1pdm09 and prone-position ventilation was not included in the survey

*IMV* invasive mechanical ventilation, *HFNO* high-flow nasal oxygen, *ECMO* extracorporeal membrane oxygenation, *ECCO*_*2*_*R* extracorporeal carbon dioxide removal, *ICU* intensive care unit

^a^% of type A, not all influenza A viruses were subtyped

We estimated the incidence rate for influenza-associated respiratory failure in ICU-hospitalizations to be 1.95 per 100,000 person-years in the studied population (95% CI 1.51–2.39).

Six out of seven ECMO patients survived (mortality 14.3%) and one out of two patients treated with ECCO_2_R survived. In general, mortality among patients treated with extracorporeal respiratory support was 22.2%. Detailed information on ECMO and ECCO_2_R patients is presented in Table 3.

**Table 3:** Characteristics of patients treated with ECMO or ECCO_2_R

	Total number	Gender, male (%)	Age, years, mean (range)	SOFA score, median (range)	Survivors, n (%)
ECMO	7	6 (87.7)	47.4 (19–62)	12 (9–13)	6 (85.7)
ECCO_2_R	2	2 (100)	60 (51–69)	8 (8)	1 (50)

*SOFA* sequential organ failure assessment, *ECMO* extracorporeal membrane oxygenation, *ECCO*_*2*_*R* extracorporeal carbon dioxide removal

Upon admission to the ICU, the following bacterial pathogens were cultured from the airways of influenza patients: *Staphylococcus aureus*, *Streptococcus pneumoniae*, *Acinetobacter baumannii*, *Pseudomonas aeruginosa*, *Klebsiella pneumoniae*, *Klebsiella oxytoca*, *Escherichia coli* and *Mycoplasma pneumoniae*. Based on the data provided by one university hospital, bacterial pathogens were found in seven (63.6%) out of 11 ICU patients with influenza. 36.4% of patients, for whom a multiplex PCR test was performed, were also positive for other viruses than influenza: parainfluenza virus, human coronavirus 229E and respiratory syncytial virus. In one ICU galactomannan tests were also performed on admittance and they were positive in two (18%) out of 11 influenza patients. *Aspergillus fumigatus* was cultured from the bronchial aspirates in those patients.

### Influenza in hospitals

Certified tests routinely employed for influenza diagnostics were used. In 15 hospitals influenza tests were performed at the hospital-based laboratory, where rapid antigen tests and immunoassays were most commonly performed. Influenza type A was detected in 544 (95.8%) hospitalized patients and type B in 24 (4.2%). The subtype A/H1N1pdm09 was detected in 241 patients, but not all influenza A viruses were subtyped (Table 4). The number of confirmed influenza infections (200 patients) was much higher in the hospital with the laboratory capacity to perform a molecular assay with multiplex PCR. In this hospital the influenza A/H1N1pdm09 virus was detected in 152 patients (76%) and 11 of them (5.5%) were treated in the ICU.

**Table 4:** Number of influenza patients in intensive care units (ICUs) and hospitals with regard to the type of virus detected

Type of virus	ICUs n = 76	Hospitals n = 568
A^a^, n (%)	35 (46)	303 (53.4)
A/H1N1pdm09, n (%)	40 (52.7)	241 (42.4)
B, n (%)	1 (1.3)	24 (4.2)

Data from 21 ICUs and 15 hospitals

^a^Not all influenza A viruses were subtyped

## Discussion

We report the results of this survey that enrolled all ICUs in two neighbouring voivodeships in south-west Poland with a population of 3.9 million inhabitants, accounting for 10% of the entire population of Poland. The majority of ICUs (63.6%) reported patients with laboratory confirmed influenza during the 2018/2019 season. In the period from 1 November 2018 to 31 March 2019 there were a total of 76 influenza patients treated in 21 ICUs, a number that posed a significant burden to the combined 182 bed capacity of those units. The mean age (62 years) and mortality (44.7%) of these patients did not differ from the average age and mortality of patients in all ICUs in Poland (63.1 years and 42%, respectively) [6] and mortality is consistent with the SOFA score predicted mortality [7]. The median SOFA score of 11 indicates dysfunction/failure of at least three organs, which predicts mortality of more than 40%, as determined in a retrospective study by Bingold on 23,795 patients [8]. In a study by Beumer et al., the ICU mortality of influenza patients was 38%, as compared to 44.7% in our study, but their patients were younger than the patients in our study (the mean age was 53 years, vs 62 years, respectively), as 16% of the patients were pediatric patients [2]. A high mortality rate of 36% in mechanically ventilated influenza ICU cases was also reported by Ludwig et al. in a study based on large, German healthcare-claim data from 2017–2019 [9].

The incidence rate of influenza-associated acute respiratory failure found in our study was high and indicates that influenza can be an important contributor to respiratory failure hospitalization. In a cohort study from 2003 through 2009 using large hospitalization databases and influenza surveillance data for Arizona, California, and Washington the incidence of influenza-associated acute respiratory failure was 2.7 per 100,000 person-years [10], slightly higher than was found in our study. The incidence rate of influenza-associated respiratory failure found in our study could be underestimated compared with the rest of Poland, as the incidence of influenza and the number of hospitalizations of influenza cases in the general population in Poland is 50% higher [11].

The result of our survey shows that there were 34 deaths of patients with laboratory confirmed influenza in the ICUs in the studied regions. According to the National Institute of Public Health–National Institute of Hygiene (NIH) reports, during the 2018/2019 season the number of influenza and influenza-like infections in Poland was 4.5 million and the number of deaths with laboratory confirmed influenza was 150 (0.003%), compared to 5.4 million and 47 in the previous season, respectively [4]. Only 10 out of 150 deaths with laboratory confirmed influenza were reported by the NIH in the Lower Silesia and Opole voivodeships, a number that contradicts the 34 deaths found in our survey, indicating that the total number of influenza deaths in the population is markedly underreported and might be at least three times higher, notably considering that only ICU patients were enrolled in the survey. Underestimation as a consequence of undercoding of hospitalized influenza cases is a recognized shortcoming of influenza surveillance systems reported also by others [12, 13]. The number of deaths was the highest since the influenza pandemics in 2009 and might be attributed to the high prevalence of the A/H1N1 influenza strain in the 2018/2019 season, responsible for 77.5% of all infections [4]. Since there are no data available about the mortality of patients with influenza in Polish ICUs, the results of our survey fill an important gap in knowledge about influenza epidemiology.

The NIH reports show that during the 2018/2019 influenza season the dominant strain in Poland was A/H1N1pdm09. Indeed, in two tertiary hospitals in which molecular tests were routinely performed, 20 out of 23 patients admitted to the ICUs who tested positive for influenza had the A/H1N1pdm09 influenza strain. Out of all the 76 ICU patients with influenza, only one had type B influenza.

In all patients invasive mechanical ventilation (IMV) was used, except one, where intubation was avoided by means of high flow nasal oxygen therapy (HFNO). The limited use of HFNO in the surveyed cohort might be due to low availability of HFNO equipment in 2019. HFNO may be an effective alternative to intubation in selected patients, as it provides the PEEP effect, improves oxygenation, reduces CO_2_ rebreathing and minute ventilation and decreases the effort of breathing [14]. In patients in the FLORALI trial with PaO_2_:FiO_2_ ratio ≤ 200 the rate of intubation was significantly lower in the HFNO group [15].

Influenza pneumonia can rapidly progress into life-threatening respiratory failure. In patients with refractory hypoxemia, despite invasive mechanical ventilation, a number of rescue therapies is available. Ventilation in the prone position in cases of moderate-to-severe ARDS is associated with reduced mortality and is currently recommended [16]. This maneuver was used in as high as 21% of mechanically ventilated influenza patients in our study and a similar rate was reported by other researchers [2, 17, 18]. Extracorporeal therapies provide substantial support to gas exchange in native lungs, thus facilitating protective lung ventilation and reducing the risk of ventilator-induced lung injury. ECMO is a complex and resource-demanding technique with availability limited to selected centers; nevertheless, 9.2% of IMV patients in our study received ECMO, which is consistent with data from a large German study that reports ECMO treatment in 7% of mechanically ventilated patients with influenza [9]. Only one patient died in the ECMO group. The low mortality of patients treated with ECMO in our study can probably be attributed to lower age compared to other patients, even though there were higher SOFA scores. Of note was the unexpectedly high prevalence of male patients (88.9%) in the group treated with extracorporeal techniques, lending support to the observation that female sex is a protective factor against the development of severe ARDS [19], although this is not in agreement with other studies [20]. ECMO is used as a rescue therapy in refractory severe respiratory failure and is associated with better outcome in influenza patients according to predictive survival models [3], which proved true in our study as well.

Influenza is known to predispose the host to secondary bacterial, viral or fungal infections [21–23]. In a multicenter study in Spain the rate of bacterial coinfections was reported to be 15 to 21% in influenza patients admitted to the ICU [17] and coinfections were evident in 55.6% of ICU-admitted patients in a study by Beumer et al. from the Netherlands [2]. Bacterial coinfection is also known to occur in 30 to 50% of adults with viral community-acquired pneumonia (CAP) [24]. Our survey shows that several bacterial pathogens, both Gram-positive and Gram-negative, were found in the airways of influenza patients admitted to the ICU.

A parainfluenza virus, coronavirus and respiratory syncytial virus were found along with the influenza virus in four out of 11 patients in the ICU where a multiplex PCR test was routinely performed. Three out of four of those patients died, possibly due to severe other co-morbidities. The significance of detecting other viruses along with an influenza virus is debatable. A positive association was found between influenza A/H1N1pdm09 and other viruses and the risk of hospitalisation, although without statistical significance in the Goka et al. study [25]. Marcos et al. reported longer hospitalisation of patients with an A/H1N1pdm09 infection and coinfection with other respiratory viruses [26].

The galactomannan test was performed in only one ICU. It was positive in two patients and the result was confirmed by a culture of the bronchial aspirate. Both patients received antifungal treatment. Influenza is a known factor predisposing the host to aspergillosis by breaking the bronchial mucosa, affecting mucociliary clearance, stimulating the secretion of interleukins and changing the Th1/Th2 balance [27]. A high rate of pulmonary aspergillosis in patients with severe influenza admitted to the ICU was diagnosed in a group of 432 patients in a multicenter cohort study by the Dutch-Belgian Mycosis Study Group [22].

## Strengths and limitations of the study

The study included only adult patients and the results do not apply to pediatric populations. Participating hospitals were not homogenous, patient management was not standardized and there were variations in the level of care and diagnostic modalities between centers. Due to the voluntary participation of hospitals some answers were incomplete; in particular, the SOFA score was missing from two ICUs (five patients).

The results of our study likely underestimate the incidence of influenza-associated respiratory failure in the general population of Poland, which in fact might be slightly higher. There is an urgent need to establish a national intensive care registry in Poland, which would acquire knowledge and provide data for comparison.

The study reflects the real life situation of the prevalence of influenza in ICUs in Poland. The results of the survey were obtained from all ICUs in the studied regions and hospitals of all levels of care were included.

## Conclusion

This study provides important information about the incidence of influenza in ICU patients in Poland. A substantial number of influenza infections occurred in the autumn–winter season of 2018/2019 that required costly treatment in the intensive care units. Upon admission to the ICU, influenza patients had a high incidence of organ failure, reflected by a high SOFA score, and the mortality rate was 44.7%. Advanced extracorporeal respiratory techniques offer real survival opportunities to patients with severe influenza-related ARDS. The presence of coinfection should be considered in patients admitted to the ICU with influenza and respiratory failure.

## Supplementary Information

**Additional file 1: Appendix S1**: Brief characteristics of participating ICUs. **Appendix S2**: Survey on patients with confirmed influenza and respiratory failure treated in intensive care units in the period from 1 November 2018 to 31 March 2019. **Appendix S3**: List of collaborators.

## Data Availability

The datasets used and/or analysed during the current study are available from the corresponding author on reasonable request.

## Abbreviations

ARDS: Acute respiratory distress syndrome
ECCO_2_R: Extracorporeal carbon dioxide removal
ECMO: Extracorporeal membrane oxygenation
HFNO: High flow nasal oxygen
ICU: Intensive care unit
IMV: Invasive mechanical ventilation
NIH: National Institute of Hygiene
PCR: Polymerase chain reaction
PEEP: Positive end-expiratory pressure
SOFA: Sequential organ failure assessment score
