# Supplementary material for: Influenza in patients with respiratory failure admitted to intensive care units in Poland and the use of extracorporeal respiratory support: a survey-based multicenter study
Source: BMC Infect Dis. 2021 Sep 15;21:954. doi: 10.1186/s12879-021-06672-w (PMC8441232; doi:10.1186/s12879-021-06672-w)
Supplement: Supplementary file 1 — Additional file 1: Appendix S1: Brief characteristics of participating ICUs. Appendix S2: Survey on patients with confirmed influenza and respiratory failure treated in intensive care units in the period from 1 November 2018 to 31 March 2019. Appendix S3: List of collaborators. [file 12879_2021_6672_MOESM1_ESM.docx]

**Additional file 1 Content**

**Appendix S1:** Brief characteristics of participating ICUs.

**Appendix S2:** Survey on patients with confirmed influenza and respiratory failure treated in intensive care units in the period from 1 November 2018 to 31 March 2019.

**Appendix S3:** List of collaborators.

**Appendix S1:** Brief characteristics of participating ICUs.

12 ICUs - level 1 hospitals*, 8 ICUs - level 2 hospitals, 5 ICUs - level 3 hospitals, 5 ICUs - national level hospitals (including 3 university hospitals), 2 ICUs - single specialty level hospitals (oncology, pulmonology). ICU bed capacity range: from 4 to 26, in total 255 beds.

*Hospital network levels as desrcibed in: Dubas-Jakóbczyk K, Kowalska-Bobko I, Sowada C. The 2017 reform of the hospital sector in Poland - The challenge of consistent design. Health Policy. 2019 Jun;123(6):538-543. doi: 10.1016/j.healthpol.2019.03.013.

**Appendix S2:** Survey on patients with confirmed influenza and respiratory failure treated in intensive care units in the period from 1 November 2018 to 31 March 2019.

Please answer at least the first question. If data on the remaining points cannot be obtained, please tick "I don't know".

**Q1.** Number of patients diagnosed with influenza treated in your ward.

**Q2.** What was the strain of influenza virus (please provide the number of cases)? Type A, type A/H1N1, I don’t know.

**Q3.** What was the outcome of these patients? Died, survived, I don’t know.

**Q4.** Respiratory therapy. Please state the most advanced that was used (number of patients): ECMO, ECCO2R, IMV, NIV, HFNO, oxygen mask/nasal prongs, prone position, I don’t know.

**Q5.** Is influenza virus diagnostics performed on site in a hospital laboratory? Yes, no, I don’t know.

**Q6.** Have other respiratory pathogens been isolated in these patients on admission to the ICU? Yes, no, I don’t know. If yes, please specify.

**Q7.** Were tests performed for the presence of the Aspergillus antigen (galactomannan)? Yes, No, I don’t know.

If yes, in how many patients was it positive?

**Q8.** What was the number of patients with positive culture of Aspergillus spp from the airways? Bronchial aspirate, BAL, miniBAL, I don’t know.

**Q9.** What was the total number of positive tests for influenza and influenza A/H1N1 in all patients admitted to the hospital from 1 November 2018 to 31 March 2019 (based on data obtained from the hospital laboratory).

**Q10.** For influenza patients, please provide gender, age and SOFA score on admission to the ICU.

**Appendix S3:** List of collaborators

Marian Jurkiewicz, Dolnoslaskie Centrum Chorob Pluc (Wroclaw); Bernard Zajac, Wojewodzki Szpital Specjalistyczny (Wroclaw); Andrzej Czyrek, Wojewodzki Szpital Specjalistyczny im.Gromkowskiego (Wroclaw); Piotr Ptaszynski, Dolnoslaski Szpital Specjalistyczny im. T. Marciniaka (Wroclaw); Gabriela Kolodziejczyk, Dolnoslaskie Centrum Onkologii (Wroclaw); Anita Gabrys-Lewsza, Szpital MSWiA (Wroclaw); Alina Rahman, Wojewodzkie Centrum Szpitalne Kotliny Jeleniogorskiej (Jelenia Gora); Elzbieta Dutkiewicz, ZOZ w Boleslawcu (Boleslawiec); Beata Mackowiak, Wojewodzki Szpital Specjalistyczny w Legnicy (Legnica); Bernadeta Tulaza, Regionalne Centrum Zdrowia EMC (Lubin); Joanna Pikul, Specjalistyczny Szpital im. Sokolowskiego (Walbrzych); Andrzej Piegza, Regionalny Szpital Specjalistyczny Latawiec w Swidnicy (Swidnica); Malgorzata Wysocka, Glogowski Szpital Powiatowy (Głogow); Witold Korycki, Wielospecjalistyczny Szpital w Zgorzelcu (Zgorzelec); Ryszard Tokarczuk, Szpital im. Sw. Jadwigi Slaskiej w Trzebnicy (Trzebnica); Mariola Maciaszek, Powiatowe Centrum Zdrowia w Kamiennej Gorze (Kamienna Gora); Marcin Karasinski, Powiatowe Centrum Zdrowia w Lwowku Slaskim (Lwowek Slaski); Henryk Szlemp, Specjalistyczne Centrum Medyczne im. Sw. Jana Pawła II (Polanica Zdroj); Joanna Mazerska, Luzyckie Centrum Medyczne w Lubaniu (Luban); Ewa Maziarz-Libionka, Szpital "Miedziowe Centrum Zdrowia" S.A. (Lubin); Irena Ziniewicz-Gryka, Szpital Milickie Centrum Medyczne (Milicz); Dariusz Grzegorczyk, Szpital Powiatowy w Klodzku (Klodzko); Elzbieta Krolicka-Garbino, Powiatowy Zespol Szpitali w Olesnicy (Olesnica); Agnieszka Dyla, Szpital Powiatowy w Olawie (Olawa); Maciej Gawor, University Hospital in Opole (Opole); Janusz Molinkiewicz, SP ZOZ MSWiA (Opole); Gabriela Werner-Osyra, Szpital Powiatowy w Olesnie (Olesno); Jerzy Kilijan, Szpital Zespolony w Kedzierzynie-Kozlu (Kedzierzyn-Kozle); Malgorzata Labuz-Margol, Szpital Powiatowy w Nysie (Nysa); Zbigniew Brachaczek, Szpital Powiatowy w Strzelcach Opolskich (Strzelce Opolskie).
